# The Efficacy and Safety of Apatinib Plus Camrelizumab in Patients With Previously Treated Advanced Biliary Tract Cancer: A Prospective Clinical Study

**DOI:** 10.3389/fonc.2021.646979

**Published:** 2021-04-12

**Authors:** Dongxu Wang, Xu Yang, Junyu Long, Jianzhen Lin, Jinzhu Mao, Fucun Xie, Yunchao Wang, Yanyu Wang, Ziyu Xun, Yi Bai, Xiaobo Yang, Mei Guan, Jie Pan, Samuel Seery, Xinting Sang, Haitao Zhao

**Affiliations:** ^1^ Department of Liver Surgery, State Key Laboratory of Complex Severe and Rare Disease, Peking Union Medical College Hospital, Chinese Academy of Medical Sciences and Peking Union Medical College, Beijing, China; ^2^ Department of Medical Oncology, Peking Union Medical College Hospital, Chinese Academy of Medical Sciences and Peking Union Medical College, Beijing, China; ^3^ Department of Radiology, Peking Union Medical College, Chinese Academy of Medical Sciences and Peking Union Medical College, Beijing, China; ^4^ Department of Humanities and Social Sciences, Peking Union Medical College, Chinese Academy of Medical Sciences and Peking Union Medical College, Beijing, China; ^5^ Faculty of Health and Medicine, Division of Health Research, Lancaster University, Lancaster, United Kingdom

**Keywords:** apatinib, camrelizumab (SHR-1210), advanced biliary tract cancer, combination therapy, PD-1/L1 blockade, target therapy, immunotherapy, cholangiocarcinoma

## Abstract

**Background:**

PD-1/L1 inhibitor-based immunotherapy is currently under investigation in biliary tract cancer (BTC). Apatinib combined with camrelizumab has achieved promising results in various tumor types. The aim of this study was to assess the safety and efficacy of apatinib plus camrelizumab for advanced biliary tract cancer patients who have received previously treatments.

**Methods:**

This prospective, non-randomized, open-label trial was conducted at Peking Union Medical College Hospital (PUMCH). All included patients received apatinib orally at 250 mg per a day and camrelizumab intravenously at 200 mg every three weeks until disease progression or intolerable toxicity occurred. Efficacy was evaluated based on the Response Evaluation Criteria in Solid Tumors RECIST Version 1.1 (RECIST 1.1). Adverse events (AEs) were assessed by the National Cancer Institute Common Terminology Criteria for Adverse Events (CTCAE version 4.0).

**Results:**

A total of 22 patients were consecutively enrolled from 1st December, 2018 until 1st August, 2020. Among 21 patients for whom we could conduct efficacy evaluations, no patients achieved a complete response (CR), 4 patients (19%) achieved partial response (PR), and 11 patients had stable disease with a disease control rate of 71.4%. The median overall survival was 13.1 months (95% CI, 8.1-18.2), and the median progression-free survival was 4.4 months (95% CI, 2.4-6.3). All patients experienced treatment related AEs, and grade 3 or 4 AEs occurred in 14 (63.6%) of 22 patients. No treatment related deaths were observed.

**Conclusions:**

This is the first report focusing on the efficacy and safety of camrelizumab plus apatinib in pretreated biliary tract cancer patients. The finding suggests this regimen has favorable therapeutic effects with relatively manageable toxicity. Further trials with a control arm are required to investigate.

**Clinical Trial Registration:**

identifier NCT04642664.

## Introduction

Biliary tract cancers (BTCs) are a heterogeneous group of cancers derived from the epithelial cells lining the biliary tree, which generally divided into intrahepatic and extrahepatic cholangiocarcinomas (ICC, ECC) and gallbladder cancers (GBC) (1). Even though BTC are traditionally regarded as rare malignant neoplasms, it is the second most common primary liver tumor and accounts for approximately 10%-15% of all hepatobiliary malignancies (2). Consistent with other gastrointestinal neoplasms, radical surgery with negative resection margins is the only potentially curative therapy. However, approximately 60-70% patients are diagnosed at late disease stages and are ineligible for surgical resection (3). Moreover, the treatment regimens for advanced BTC patients are extremely scarce with limited efficacy. Only a few chemotherapies including gemcitabine plus cisplatin or another platinum derivative have been approved as first-line interventions, with only modest efficacy, and there are no consensus standard regimens for second-line and later therapy (4, 5). Given these factors, effective treatments are needed to fill in gaps in current BTC treatment approaches and prolong the survival of patients (6).

Programmed cell death protein 1 or ligand 1 (PD-1/L1) blockades are relatively novel therapeutics which have been tested for a variety of tumors and have found to have robust, durable antitumor activity. However, the efficacy of PD-1/L1 inhibitor monotherapy in gastrointestinal malignancies is not ideal owing to the complex tumor microenvironment, for example the presence of abundant fibrotic stroma that surrounds and infiltrates the tumor structures can hinder the antitumor functions of T cells (7). For BTCs, the objective response rate (ORR) of PD-1 blockade monotherapy is approximately 4%-18%, although accumulating findings demonstrate patients with cholangiocarcinoma with specific pathological and genomic characteristics might benefit from immunotherapy (8, 9). As such, immunotherapeutic research in this field has tended to focus on seeking combinations which destroy stroma while promoting tumor antigens presentation and enabling immune recognition (7). With the substantial progress in research regarding the tumorigenesis and genetic landscape of BTCs, targeted drugs including multiple small-molecule tyrosine kinase inhibitors are also being actively explored. Current evidence indicates that 53% intrahepatic cholangiocarcinoma harbor vascular endothelial growth factor (VEGF) overexpression which is related to poorer prognosis (10). Preclinical models appear to suggest that agents targeting VEGF, FEGF, EGFR and other signaling pathways can convert the tumor microenvironment and reprogram the immune responses to suppress tumorigenesis (11).

Apatinib, a multitarget tyrosine kinase inhibitor (TKI) that selectively inhibits VEGFR-2, has proven beneficial for various solid tumors including gastric cancer and hepatocellular carcinoma (12). A small sample study of patients with unresectable intrahepatic cholangiocarcinoma revealed that apatinib has manageable toxicities with a median progression-free survival (PFS) of 4.5 months and overall survival (OS) of 6.5 months (13). Meanwhile, another study include patients with primary liver cancer showed that apatinib achieved 16% ORR (14). Camrelizumab (SHR-1210), a PD-1 inhibitor, has been shown to block the binding of PD-1 to PD-L1 and consequently inhibit the immune escape of tumour cells. Therefore, considering the potential synergistic efficacy of targeted therapy combined with immune checkpoint inhibitor, apatinib plus camrelizumab might be a potentially effective combination for various tumors (15). For patients with advanced HCC, apatinib combined with camrelizumab achieved a 34.3% objective response as the first-line and 22.5% as the second-line therapy (16). Similar results were also observed in osteosarcoma, gastric cancer, advanced triple-negative breast cancer and a variety of other tumors (17–19). Nevertheless, thus far, no studies have reported results regarding this regimen in patients with BTC. As such, we conducted this prospective clinical trial to evaluate the efficacy and safety of apatinib in previously treated patients with advanced BTC in hopes of providing an alternative treatment regimen.

## Methods

### Study Design and Participants

This was a prospective, single institution, open-label, nonrandomized trial designed to evaluate the efficacy and safety of apatinib in combination with camrelizumab for advanced BTC patients. The study protocol adhered to the principles of the Declaration of Helsinki and was approved by the Institutional Review Board and Ethics Committee of Peking Union Medical College Hospital (PUMCH-JS-2160). The clinical trial is registered at ClinicalTrials.gov (NCT04642664).

All patients were required to provided written informed consent before participating. Patients were enrolled from 1st December, 2018 until 1st August, 2020. The primary inclusion criteria were patients older than 18 years with either histologically or cytologically confirmed BTC diagnosis, including ICC, ECC and GBC. The patients had at least one measurable tumor lesion at baseline per the Response Evaluation Criteria in Solid Tumors, version 1.1 (RECIST v1.1) and had received at least a previous systemic anti-tumor therapy. Patients had Child Pugh A or B liver function status (score ≤7) and presented with an Eastern Cooperative Oncology Group performance status (ECOG PS) value of 0-2.

The exclusion criteria mainly included intolerance to apatinib or PD-1 blockade, life expectancy of ≤3 months and inadequate organ function including Child-Pugh liver function class C, active or prior autoimmune disease, concurrent use of immunosuppressive medicaments and other contraindicators associated with apatinib or camrelizumab. Patients with severe esophageal varices or those who presented with positive fecal occult blood were also excluded. The detailed study criteria are available in the Supplement.

### Assessment of Efficacy and Treatment Related Adverse Events (AEs)

Patients received apatinib orally at 250 mg per day, irrespective of body mass. During treatment, apatinib could be reduced to a half dose or administered once every other day considering the grade of treatment-related AEs. Camrelizumab was administered intravenously at a dosage of 200mg over 30 minutes every 3 weeks. The interruption period of camrelizumab was no longer than six weeks. All patients continued combination treatment until disease progression, unacceptable toxicity or discontinuation for any reasons.

Tumor were assessed using enhanced computed tomography, magnetic resonance imaging (MRI) or other available imaging technologies at baseline and every 4-8 weeks until disease progression or treatment discontinuation. The therapeutic efficacy assessment included the ORR, disease control rate (DCR), PFS, OS and clinical benefit rate (CBR) according to RECIST 1.1. The CBR was defined as the proportion of patients who achieved a radiologically confirmed objective response (CR or PR) or those who encountered stable disease longer than 6 months. Patients who had progressive disease could continue treatment, if the investigator determined patients would benefit from continuing. When patients discontinued treatment, follow-up was conducted every month to assess survival.

During the observation period, tolerability and toxicity were collected in detail and assessed according to the National Cancer Institute Common Terminology Criteria for Adverse Events version 4.0 (CTCAE 4.0). Patients who received at least one dose of camrelizumab plus apatinib were included in the safety assessment set, and AEs were collected until 30 days after the last dose. According to the study protocol, when grade 3 or more severe AEs occurred, dose reduction was implemented or a temporary interruption commenced until symptoms subsided to pharmaceutically manageable grades 1 or 2. Patients with grade 3 or more severe AEs were followed up to 90 days after the lase dose or until the new anticancer treatment.

### Multivariate Analysis of Characteristics and Therapeutic Response Predictions

Baseline characteristics including age, sex, hepatitis B virus (HBV) infection status, ECOG performance score, histopathological grade, site of metastases and number of previous treatments were analyzed using multivariate method to explore the potential factors affecting PFS or OS. Patients who were evaluated as having stable disease were further divided into two groups: those with a reduction in tumor size and those with an increase in tumor size. Carbohydrate antigen 19-9 (CA19-9) values were recorded before and after treatment within first evaluation period to develop response predictions for tumor size changes. On the basis of the previous studies, patients who are Lewis-antigen-negative (7% of the general population) have undetectable CA 19–9 levels. If patient’s CA199 level was within the normal range before and after treatment, we excluded these patients from the further analysis.

In addition, we assessed PD-L1 expression in this BTC population. Tumor tissue samples for analyzing PD-L1 expression were collected from patients. Preserved tumor specimens were formalin-fixed, paraffin-embedded (FFPE) and then cut into 4-5 μm thick sections for further staining. The primary antibody used was anti-PD-L1 (IHC 22C3 pharmDx, Dako North America, Agilent Technologies). Specimens in which PD-L1 was expressed in 1% or more tumor cells and 1% or more tumor-associated immune cells were defined as positive for PD-L1 expression.

### Statistical Analysis

Baseline data were calculated and presented as the means with corresponding standard deviations or as simple numbers and percentages. Categorical variables in the different subgroups were compared using the Fisher’s exact test. The Kaplan-Meier method was applied to generate PFS and OS curves, and the log-rank test was used to compare curves from different PD-L1 expression subgroups. Two tailed P values of less than 0.05 were considered to be indicative of statistical significance. Sensitivity was calculated as the number of correctly classified divided by total true decreased individuals, and specificity was calculated as the number of true negatives divided by all non-decreased individuals. Univariate and multivariate analysis of baseline characteristics for overall survival and progression-free survival were conducted by Cox proportional hazards regression. All statistical analyses were performed using IBM SPSS 22.0 and R software (version 3.6.5).

## Results

### Patients Characteristics

**Figure 1:**
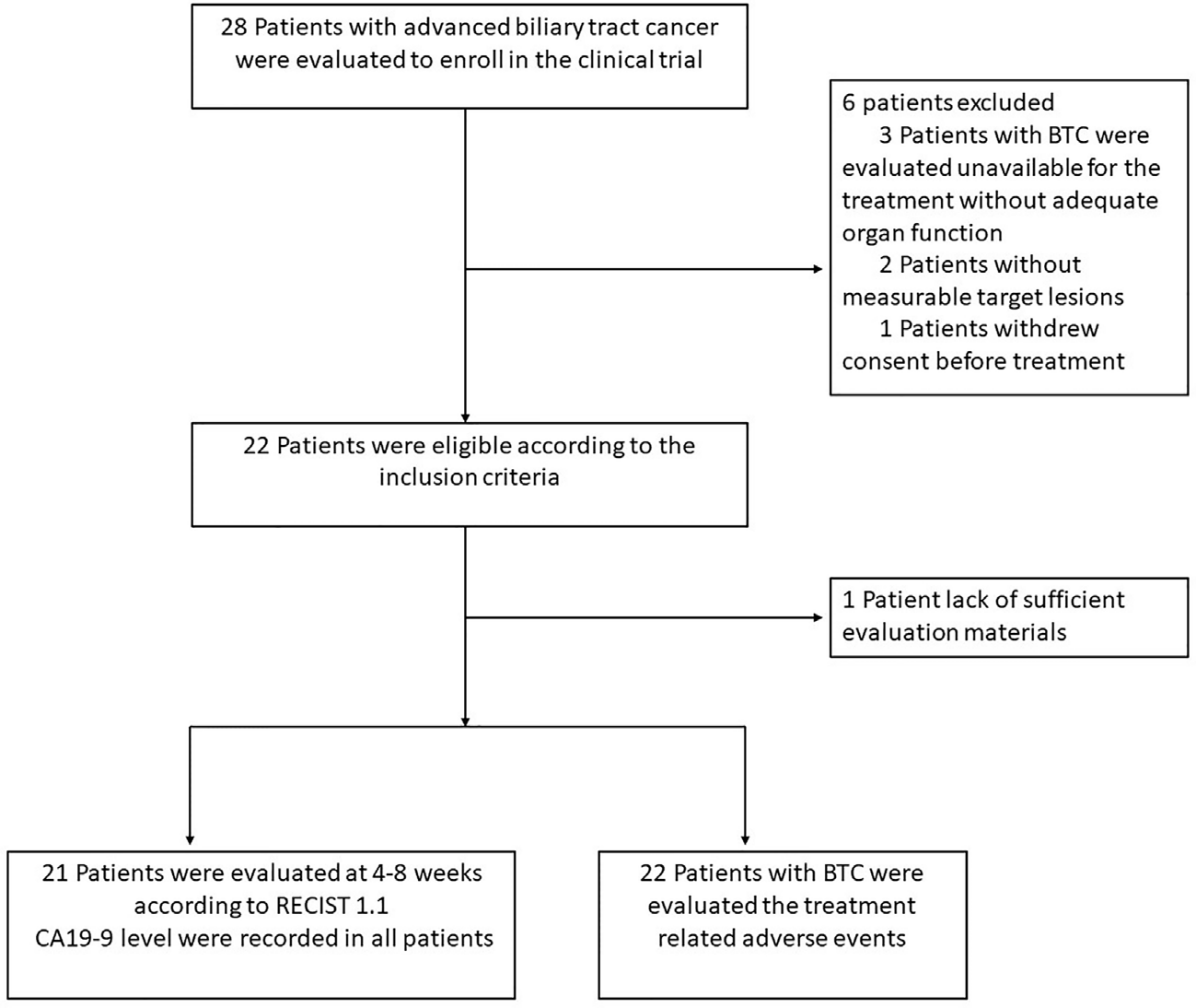
Flow diagram of study population.

From December 01, 2018 to August 01, 2020, the study totally evaluated 28 patients and six patients were excluded according to the inclusion criteria; at last, 22 patients were consecutively enrolled for drug administration, one patient was excluded from the study due to the lack of necessary evaluations (**Figure 1**). The median patient age was 58 years (range, 39-72) and 11 patients (52%) were male. Most patients (16 [76.2%]) had an Eastern Cooperative Oncology Group performance status of 1 and 7 patients (33.3%) presented with HBV infection. Fifteen patients (71%) had intrahepatic cholangiocarcinoma, 4 patients (19%) had extrahepatic cholangiocarcinoma, and 2 (9%) patients had gallbladder cancer. Regarding the histopathological grade of cholangiocarcinoma, 4 patients (19%) had undetermined grade, eight patients (38.1%) had poorly differentiated tumors, seven patients (33.3%) had moderately differentiated tumors, and two patients (9.5%) had well differentiated tumors. Of the 21 patients, 20 patients (95.2%) presented with metastatic disease, and 12 patients (57.2%) experienced recurrence after radical resection. Regarding the site of metastases, most patients (17, 80.9%) had intrahepatic or lymph nodes metastases, and the lung metastases occurred in 8 patients (38.1%). Twenty patients (95.2%) underwent systemic chemotherapy, and 10 patients (47.6%) received at least two kinds of treatment regimens. In addition, CA19-9 levels exceeding 150 ng/ml were observed in twelve patients (57.1%) (**Table 1**).

**Table 1:** Patient baseline demographics and disease characteristics.

	ALL (n=21)
Age (median, range)	60 (39-72)
Sex (female: male)	10:11
BMI (mean (SD))	23 (3.5)
Hepatitis (HBV) infection n, (%)	7 (33.3)
ECOG performance n, (%)	
0	2 (9.5)
1	16 (76.2)
2	3 (14.3)
Tumor subtype n, (%)	
ICC	15 (71.4)
ECC	4 (19.0)
GBC	2 (9.5)
Histopathologyical grade	
Well differentiated (low grade)	2 (9.5)
Moderately differentiated (intermediate grade)	7 (33.3)
Poorly differentiated (high grade)	8 (38.1)
Unable to determine	4 (19.0)
Extent of disease n, (%)	
Metastatic	20 (95.2)
Recurrent	12 (57.2)
Site of Metastases n, (%)	
Intrahepatic	17 (80.9)
Lymph nodes	17 (80.9)
Lung	8 (38.1)
Others	5 (23.8)
Number of previous treatment regimens n, (%)	
1	11 (52.4)
≥2	10 (47.6)
Previous treatment regimens n, (%)	
Systemic chemotherapy	20 (95.2)
Targeted therapy	6 (28.6)
Regional radiotherapy or ablation	5 (23.8)
Transarterial chemoembolization	5 (23.8)
CA19-9> 150 n, (%)	12 (57.1)
Size of target lesion (mean (SD)	7.3 (3.5)

BMI, body mass index; ECOG, Eastern Cooperative Oncology Group; HBV, hepatitis type B virus; CA19-9, carbohydrate antigen 19-9; ICC, intrahepatic cholangiocarcinoma; ECC, extrahepatic cholangiocarcinoma; GBC, gallbladder cancer.

### Assessment of Efficacy and AEs During the Entire Treatment Period

**Figure 2:**
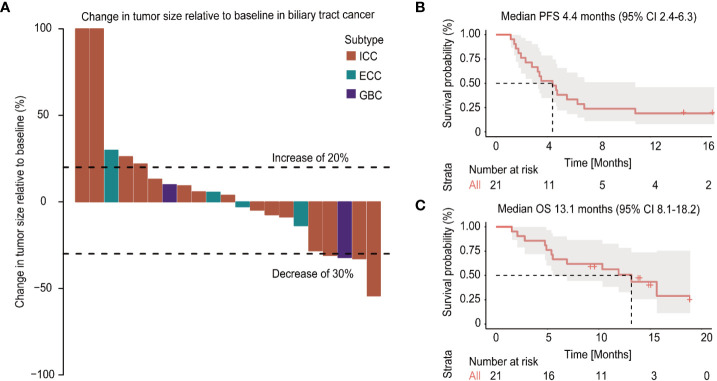
Changes in tumor burden from baseline of the response-evaluable patients and survival plot **(A)**. Kaplan-Meier curves of the progression-free survival (PFS) **(B)** and overall survival (OS) **(C)** of patients with biliary tract cancer (BTC) treated with apatinib plus camrelizumab.

Among the 21 patients who had evaluation data with imaging examination, ten patients (47%) exhibited a reduction in tumor size as the best response during the treatment period, while 11 patients (52%) exhibited an increase in tumor size, of which two patients experienced new lesions (**Figure 2**). According to the RECIST 1.1, no CR was observed, 4 patients (19%) achieved a partial response (PR) with an ORR of 19%, and 11 patients had stable disease with a DCR of 71.4% (**Table 2**). An objective response was observed in 3 of 15 patients (25%) with intrahepatic cholangiocarcinoma, and a PR was observed in one patient with gallbladder cancer. As of the data cut-off date of August 01, 2020, the median duration of follow-up was 13.4 months (IQR 11.9-14.8), the median duration of treatment was 4.9 (IQR, 3.8-5.9) months, and 4 (19%) of 21 patients were still receiving treatment (**Table 3**). In the entire cohort, the median OS was 13.1 months (95% CI, 8.1-18.2), and the median PFS was 4.4 months (95% CI, 2.4-6.3) (**Figure 1**).

**Table 2:** Clinical efficacy in BTC patients treated with apatinib plus camrelizumab.

Investigator review according to RECIST 1.1	All (n=21)
Objective response rate (%, 95% CI)	19.0% (7-40)
Complete response (n, %)	0
Partial response (n, %)	4 (19%)
Stable disease (n, %)	11 (52.3%)
Progressive disease (n, %)	6 (28.5%)
Disease control rate (%, 95% CI)	71.4% (50-86.1)
Clinical benefit rate (%, 95% CI)	33.3% (17.1-54.6)
Progression-free survival (months, 95% CI)	4.4 (2.4-6.3)
Overall survival (months, 95% CI)	13.1 (8.1-18.2)
Decreased CA19-9 predicts tumor reduction	
Se (%, 95% CI)	63.6% (35.3-84.8)
Sp (%, 95% CI)	66.7% (35.4-87.9)

BTC, biliary tract cancer; RECIST 1.1, response evaluation criteria in solid tumors, version 1.1; CA19-9, carbohydrate antigen 19-9; Se, sensitivity; Sp, specificity.

**Table 3:** Clinical characters of BTC patients treated with Apatinib plus Camrelizumab.

	All (n=21)
median Follow-up time (months, 95% CI)	13.4 (11.9-14.8)
median Treatment time (months, 95% CI)	4.9 (3.8-5.9)
Patients still receive the treatment	4/21
Patients of PD-L1 positive	4/21
Continue Treatment after Progression Disease	9/21
Continue Targeted	9/21
Continue PD-1/L1 inhibitor	7/21

BTC, biliary tract cancer.

The observed treatment-related AEs are summarized in **Table 4**. All patients experienced at least one kind of adverse event, and grade 3 or 4 AEs occurred in 14 (63.6%) of 22 patients. The most common treatment-related AEs of any grade were asthenia (15, 68.2%), decreased appetite (10, 45.5%) and hypertension (7, 31.8%). The most common grade 3 or 4 AEs were hypertension (3, 13.6%), blood bilirubin increase (3, 13.6%) and platelet count decrease (3, 13.6%). Eighteen patients (81.8%) experienced treatment interruption or modification and three patients (13.6%) discontinued combination therapy due to treatment related AEs. No deaths related to treatment were observed.

**Table 4:** Treatment-related AEs in all patients with biliary tract cancer.

	All treated patients (n=22)	
	Any grade n, (%)	Grade 3/4 n, (%)
Asthenia	15 (68.2)	1 (4.5)
Decreased appetite	10 (45.5)	
Hypertension	7 (31.8)	3 (13.6)
Increased alanine aminotransferase	7 (31.8)	2 (9.1)
Rash	7 (31.8)	2 (9.1)
Abdominal pain	7 (31.8)	
Increased blood bilirubin	6 (27.3)	3 (13.6)
Pain	6 (27.3)	1 (4.5)
Hypoalbuminemia	6 (27.3)	
RCCEP	6 (27.3)	
Increased aspartate aminotransferase	5 (22.7)	2 (9.1)
Abdominal distention	9 (40.9)	
Nausea	5 (22.7)	
Decreased platelet count	4 (18.2)	3 (13.6)
Hypothyroidism	4 (18.2)	
Vomiting	3 (13.6)	1 (4.5)
Proteinuria	3 (13.6)	
Fever	3 (13.6)	
Palmar–plantar erythrodysesthesia	3 (13.6)	
syndrome	3 (13.6)	
Digestive tract hemorrhage	2 (9.1)	2 (9.1)
Decreased leukopenia	2 (9.1)	
Diarrhea	2 (9.1)	
Decreased neutropenia	1 (4.5)	
Decreased hemoglobin	1 (4.5)	

### Subgroup Analyses and Therapeutic Response Predictions

**Figure 3:**
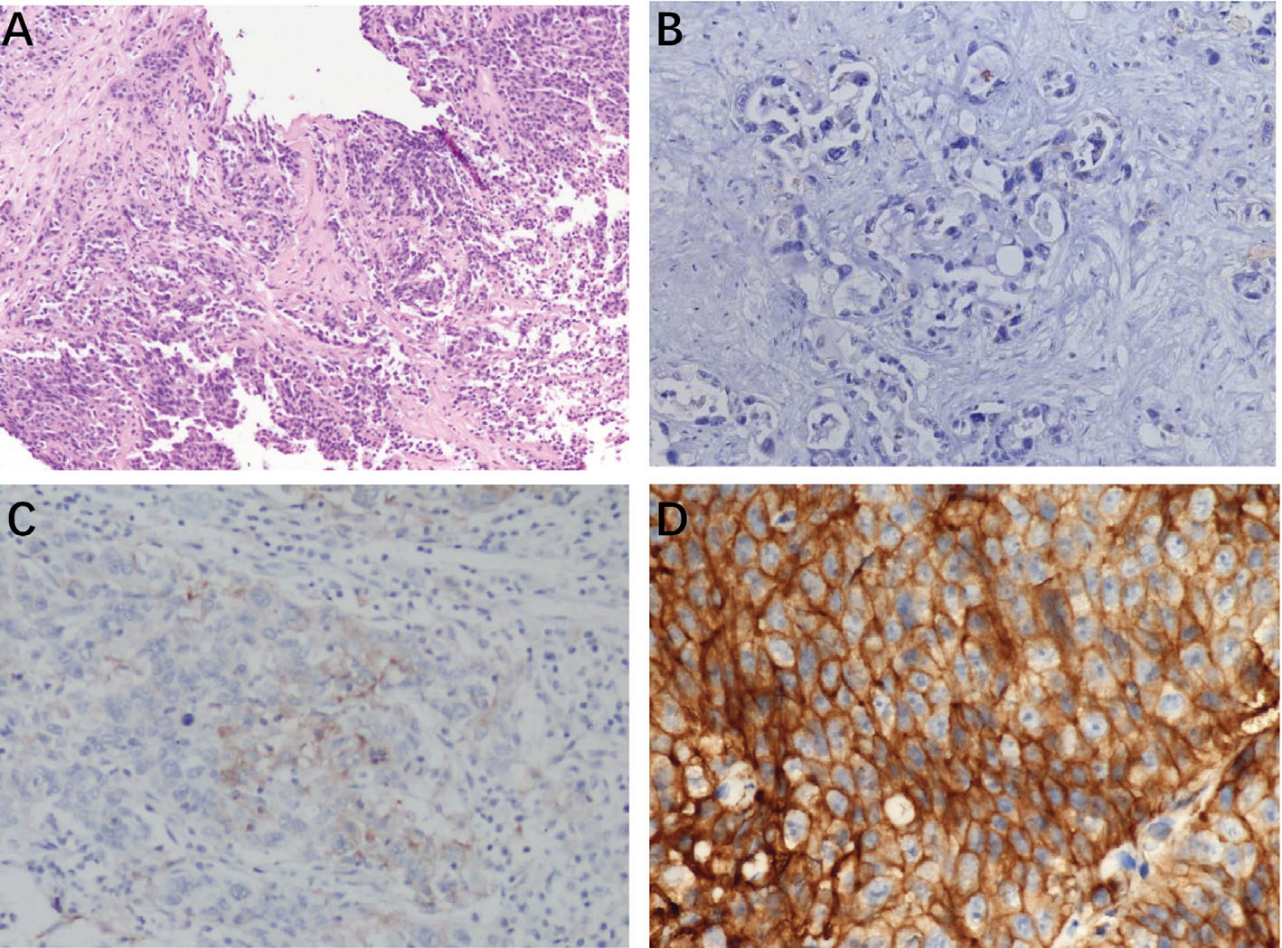
Typical photomicrographs of PD-L1 immunohistochemistry in patents’ archived pretreatment formalin-fixed and paraffin-5 embedded tumor tissue: **(A)** HE staining, 100×; **(B)**PD-L1 negative, 200×; **(C)** PDL1-stained tumor cell (3%) 100×; **(D)** positive PD-L1 expression (50%) 400×.

**Figure 4:**
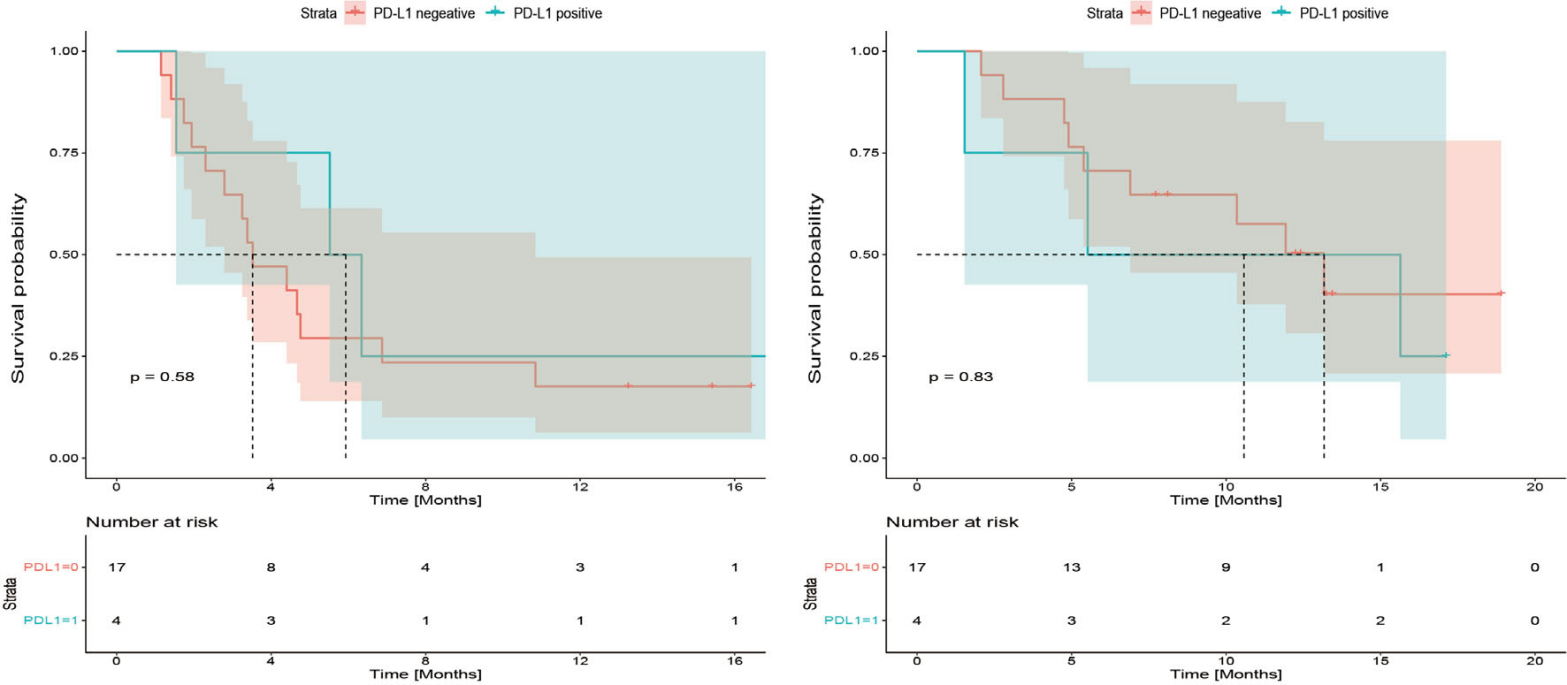
Kaplan-Meier plot for progression-free survival (PFS) and overall survival (OS) based on programmed cell death 1 ligand-1 (PD-L1) immunohistochemical expression.

**Figure 5:**
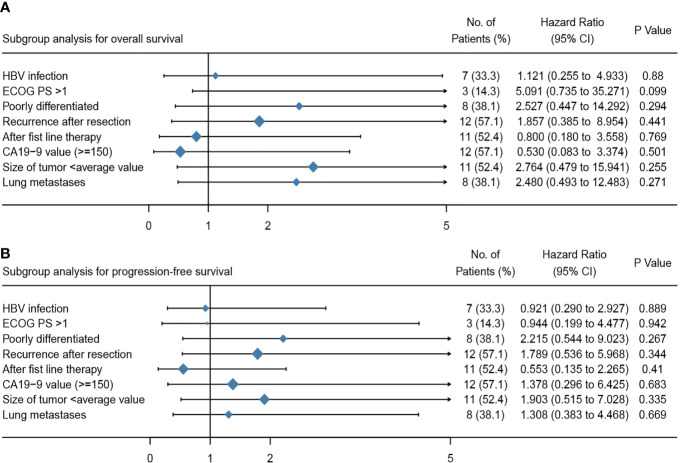
Multivariate analyses of the overall survival **(A)** and progression free survival **(B)** for patients treated with apatinib plus camrelizumab. ECOG PS, Eastern Cooperative Oncology Group performance status; CA19-9, carbohydrate antigen 19-9.

Among 21 available tumor samples, 4 patients (19%) were positive for PD-L1 staining in tumor cells or immune cells (**Figure 3**). Of the four PD-L1-positive patients, three patients experienced tumor reduction, but only one patient had experienced PR. Median PFS and OS were not significantly different (*p*=0.58, *p*=0.83) between the patients with PD-L1 expression ≥1% and those with PD-L1 expression <1% (**Figure 4**). Although the limited sample in current study, considering the heterogeneity of biliary tract cancer, we analyzed the efficacy and safety of different tumor subtypes. The results showed that there was no significant difference among intrahepatic cholangiocarcinoma, extrahepatic cholangiocarcinoma and gallbladder carcinoma regarding the ORR (χ2 = 2.666, *p*=0.264), grade 3-4 AEs (χ2 = 0.649, *p*=0.723) and PFS (*p*=0.958), OS (*p*=0.725) (**Supplement Table 1**). The Cox-regression analysis results regarding the relationship between baseline characters and PFS or OS were summarized in the forest plot (**Figure 5**) and **Supplement Table 2**. The CA19-9 serum value decreased in 10 patients (47.6%) after treatment (**Supplement Table 3**). The decrease in CA19-9 value was able to predict the tumor size reduction with a sensitivity and specificity of 63.6% and 66.7%, respectively.

## Discussion

Currently, the exploration of immune monotherapy faces many challenges in terms of various gastrointestinal malignancies. The efficacy of PD-1/L1 inhibitor monotherapy in BTC remains significantly uncertainty (ORR range 3%-22%) (20). Combining PD1/L1 inhibitor with other available anticancer therapies can improve the efficacy of immunotherapies has reached a general consensus (7). In the Makoto et al. study, only one of 30 patients who received nivolumab monotherapy achieved an objective response, with a median OS of 5.2 months and a median PFS of 1.4 months. However, in the combined therapy cohort (nivolumab and cisplatin plus gemcitabine), 11 of 30 patients achieved an objective response, the median OS and PFS were 15.4 months and 4.2 months, respectively (21). In this study, apatinib in combination with camrelizumab also showed a potent efficacy in terms of the ORR, PFS and OS and had manageable toxicity. This is the first report of this regimen in advanced BTC, and these results were superior to the previously reported efficacy of apatinib alone in BTC (13, 14).

Although camrelizumab combined with apatinib has achieved promising results in the treatment of various tumors, including hepatocellular carcinoma, osteosarcoma and triple-negative breast cancer, this regimen has not been reported in cholangiocarcinoma (16, 17, 19). Currently, most trials combining multitarget TKIs with PD-1 blockade for BTC are in the recruitment phase, and few studies have reported detailed results. A study evaluating lenvatinib plus pembrolizumab as a non-first-line treatment in 32 patients with advanced BTC demonstrated that the ORR could reach 25% with a PFS of 4.9 months (95% CI: 4.7-5.2) and OS of 11.0 months (95% CI: 9.6-12.3) (22). In this study, almost all the patients (95.2%) were previously treated with at least one kind of systemic chemotherapy, and 47.6% of patients had received two or more anticancer treatments, which demonstrated that the population of this study was not similar to that of other previous studies regarding immunotherapy for BTC. In spite of this, 19% patients in this study achieved a partial response with a favorable survival. 42.8% patients who were confirmed progressive disease continued other immunotherapy or targeted drugs, which might explain this prolonged OS.

Higher PD-L1 expression is related to the favorable efficacy of immunotherapy have been reported by several studies (7, 8). In BTCs, Kabir Mody et al. analyzed the PD-L1 expression of 652 tumors by immunohistochemistry (IHC) and found that 8.6% of specimens [GBC, 12.3% (25/203); ICC, 7.3% (27/372); and ECC, 5.2% (4/77)] had PD-L1-positive tumor cells. In addition, recently published results from a BTC patient cohort receiving immunotherapy reported a positive rate of PD-L1 expression of between 9% and 11.6% (23, 24). In our present study, PD-L1 expression was found in four patients (19%), and there was no significant difference between the PD-L1-positive and PD-L1-negative patients, which is consistent with the results for advanced biliary cancer in the Keynote 158 study (25). After careful analysis of the treatment regimens of the PD-L1 positive patients and their clinical characteristics, we found that the ECOG PS of these two patients differed and that they had both received multiline treatment, which potentially affected the efficacy of immunotherapy. The limited sample size of this study may also explain the lack of significance regarding efficacy in PD-L1 positive patients. In the Kim et al. study, nivolumab treatment in a single group of patients with refractory BTCs resulted in a 22% ORR with a median PFS of 4.0 months. Although the PD-L1 expression rate of tumor cells was 43% higher than that in previous studies, which potentially means that PD-L1 expression in BTC remains a good prognostic biomarker for immunotherapy, a more comprehensive evaluation of efficacy is required for future clinical trials (26). As reported in BTC patients undergoing chemotherapy, pretreatment CA19-9 levels and the decrease in CA19-9 after treatment are of prognostic relevance. In our study, CA19-9 may be another predictive biomarker for potentially judging the response to the current treatment. But it’s important to note that patients who are Lewis-antigen-negative (7% of the general population) have undetectable CA 19–9 levels (27, 28). In addition, although apatinib has various tumor targets, the strong inhibitory effect mainly focus on VEGFR-2. Monitoring the expression of VEGFR-2 will also contribute to the prediction and evaluation of therapeutic efficacy.

The safety profile and tolerance of apatinib combined with camrelizumab in this study were similar to those in previous studies exploring apatinib, camrelizumab or combination regimens in gastroenteric neoplasms (14). Based on previous studies regarding the AEs of apatinib, this study excluded patients who had potential risk of gastrointestinal bleeding (29). Although every patient had experienced at least one kind of treatment related adverse event, most of these AEs were grade 1-2 and were well tolerated. Moreover, there were no treatment related deaths, and 63.6% of patients reporting treatment related grade 3-4 AEs had well control after stopping drug delivery. As reported in a study regarding nivolumab monotherapy for BTC (26), among the 3-4 grade AEs in this study, hepatic toxic effects were prominent and included an increase in ALT, AST and serum bilirubin levels and a decrease in platelet counts. These results demonstrate that immunotherapy alone or in combination with targeted drugs probably leads to a decrease in liver function because certain types of treatment related AEs may be affected by disease sites (30). The targets of apatinib are VEGFR-2, PDGFRβ, SRC, c-KIT and RET, and its IC 50 is lower than that of other VEGF inhibitors, which suggest that it has more AEs than some other targeted drugs applying to liver cancer. However, this study administered the lowest dose of apatinib, 250mg, and the regimens of administration were flexible, allowing patients to take the medicine every other day, take half of the 250 mg dose or take the drug for five days and stop for two days if the patients experienced 3-4 grade AEs. This flexible regimen can effectively prevent the further escalation of AEs when apatinib is readministered. In addition, the rate of reactive cutaneous capillary endothelial proliferation (27.3%) was reduced in this study compared with that reported in previous camrelizumab monotherapy studies (67%) (31).

BTC is a group of relatively rare heterogeneous cancers. In various clinical studies, the number of patients with cholangiocarcinoma is still one of the main factors that prevent a better interpretation of the results and a more scientific design of the trial. Similar to other previous studies, the main limitation of this study was its small sample size, which may lead the bias of the multivariate analysis and subgroup analysis of tumor types. Moreover, the study was a single center-initiated clinical trial that lacked a control cohort. Up to now, most studies on immunotherapy for cholangiocarcinoma are in an exploratory stage, and no phase III clinical data have been published. In general, this study was a tentative first step to explore the safety and efficacy of apatinib combined with camrelizumab in heavily pretreated BTC patients, and offers detailed clinical trial experience that can be applied to subsequent investigations.

## Data Availability Statement

The original contributions presented in the study are included in the article/**Supplementary Material**. Further inquiries can be directed to the corresponding author.

## Ethics Statement

The studies involving human participants were reviewed and approved by Institutional Review Board and Ethics Committee of Peking Union Medical College Hospital (PUMCH-JS-2160). The patients/participants provided their written informed consent to participate in this study. Written informed consent was obtained from the individual(s) for the publication of any potentially identifiable images or data included in this article.

## Author Contributions

DW, XY, JYL, and JZL collected the data and wrote the manuscript. JM, FX, YCW, YYW, ZX, and YB helped to collect literature and participated in discussions. XBY, MG, JP, XS, and HZ designed and verified the study. SS and HZ examined the language of this study. All authors contributed to the article and approved the submitted version.

## Funding

This work was supported by the International Science and Technology Cooperation Projects (2016YFE0107100), the Capital Special Research Project for Health Development (2014-2-4012), the Beijing Natural Science Foundation (L172055 and 7192158), the National Ten-thousand Talent Program, the Fundamental Research Funds for the Central Universities (3332018032), the CAMS Innovation Fund for Medical Science (CIFMS) (2017-I2M-4-003 and 2018-I2M-3-001) and the Innovation Fund for Graduate Students of Peking Union Medical College.

## Conflict of Interest

The authors declare that the research was conducted in the absence of any commercial or financial relationships that could be construed as a potential conflict of interest.

## Abbreviations

HCC, hepatocellular carcinoma; AE, adverse event; PFS, progression free survival; HR, hazard ratio; CI, confidence interval; BCLC, Barcelona Clinic Liver Cancer; AFP, alpha-fetoprotein; HBV, hepatitis B virus; OS, overall survival; ORR, objective response rate; TTP, time-to-progression; RCT, randomized controlled trials; DCR, disease control rate; RDI, relative dose intensity; TACE, transarterial chemoembolization; HCV, hepatitis C virus; RECIST 1.1, Response Evaluation Criteria in Solid Tumours version 1.1; CR, complete response; PR, partial response; SD, stable disease; PD, progressive disease; CTCAE, common terminology criteria for adverse events; HBsAg, hepatitis B virus surface antigen; ECOG-PS, Eastern Cooperative Oncology Group performance status; ALBI, albumin-bilirubin; K-M, Kaplan-Meier; OR, odds ratios; EHS, extrahepatic spread; PVT, portal vein thrombus; MVI, macrovascular invasion; TKI, tyrosine kinase inhibitors; FGFR, fibroblast growth factor receptor; FGF, fibroblast growth factor; VEGF, vascular endothelial growth factor.

## References

[1] Razumilava N Gores GJ. Cholangiocarcinoma. Lancet (2014) 383:2168–79. 10.1016/S0140-6736(13)61903-0 PMC4069226 24581682

[2] Asrani SK Devarbhavi H Eaton J Kamath PS. Burden of liver diseases in the world. J Hepatol (2019) 70:151–71. 10.1016/j.jhep.2018.09.014 30266282

[3] Bridgewater J Galle PR Khan SA Llovet JM Park JW Patel T. Guidelines for the diagnosis and management of intrahepatic cholangiocarcinoma. J Hepatol (2014) 60:1268–89. 10.1016/j.jhep.2014.01.021 24681130

[4] Valle J Wasan H Palmer DH Cunningham D Anthoney A Maraveyas A. Cisplatin plus gemcitabine versus gemcitabine for biliary tract cancer. N Engl J Med (2010) 362:1273–81. 10.1056/NEJMoa0908721 20375404

[5] Tella SH Kommalapati A Borad MJ Mahipal A. Second-line therapies in advanced biliary tract cancers. Lancet Oncol (2020) 21:e29–41. 10.1016/S1470-2045(19)30733-8 31908303

[6] Rizvi S Khan SA Hallemeier CL Kelley RK Gores GJ. Cholangiocarcinoma - evolving concepts and therapeutic strategies. Nat Rev Clin Oncol (2018) 15:95–111. 10.1038/nrclinonc.2017.157 28994423 PMC5819599

[7] Wang D Lin J Yang X Long J Bai Y Yang X. Combination regimens with PD-1/PD-L1 immune checkpoint inhibitors for gastrointestinal malignancies. J Hematol Oncol (2019) 12:42. 10.1186/s13045-019-0730-9 31014381 PMC6480748

[8] Blair AB Murphy A. Immunotherapy as a treatment for biliary tract cancers: A review of approaches with an eye to the future. Curr Probl Cancer (2018) 42:49–58. 10.1016/j.currproblcancer.2017.10.004 29501212 PMC6178815

[9] Job S Rapoud D Dos Santos A Gonzalez P Desterke C Pascal G. Identification of Four Immune Subtypes Characterized by Distinct Composition and Functions of Tumor Microenvironment in Intrahepatic Cholangiocarcinoma. Hepatology (2020) 72:965–81. 10.1002/hep.31092 PMC7589418 31875970

[10] Sia D Tovar V Moeini A Llovet JM. Intrahepatic cholangiocarcinoma: pathogenesis and rationale for molecular therapies. Oncogene (2013) 32:4861–70. 10.1038/onc.2012.617 PMC3718868 23318457

[11] Fabris L Sato K Alpini G Strazzabosco M. The Tumor Microenvironment in Cholangiocarcinoma Progression. Hepatology (2020) 73 Suppl 1(Suppl 1):75–85. 10.1002/hep.31410 32500550 PMC7714713

[12] Scott LJ. Apatinib: A Review in Advanced Gastric Cancer and Other Advanced Cancers. Drugs (2018) 78:747–58. 10.1007/s40265-018-0903-9 29663291

[13] Hu Y Lin H Hao M Zhou Y Chen Q Chen Z. Efficacy and Safety of Apatinib in Treatment of Unresectable Intrahepatic Cholangiocarcinoma: An Observational Study. Cancer Manag Res (2020) 12:5345–51. 10.2147/CMAR.S254955 PMC7342502 32753952

[14] Zhen L Jiali C Yong F Han X Hongming P Weidong H. The Efficacy and Safety of Apatinib Treatment for Patients with Unresectable or Relapsed Liver Cancer: a retrospective study. J Cancer (2018) 9:2773–7. 10.7150/jca.26376 PMC6096375 30123344

[15] Athauda A Fong C Lau DK Javle M Abou-Alfa GK Morizane C. Broadening the therapeutic horizon of advanced biliary tract cancer through molecular characterisation. Cancer Treat Rev (2020) 86:101998. 10.1016/j.ctrv.2020.101998 32203843 PMC8222858

[16] Xu J Shen J Gu S Zhang Y Wu L Wu J. Camrelizumab in combination with apatinib in patients with advanced hepatocellular carcinoma (RESCUE): a non-randomized, open-label, phase 2 trial. Clin Cancer Res (2021) 27(4):1003–11. 10.1158/1078-0432.CCR-20-2571 33087333

[17] Liu J Liu Q Li Y Li Q Su F Yao H. Efficacy and safety of camrelizumab combined with apatinib in advanced triple-negative breast cancer: an open-label phase II trial. J Immunother Cancer (2020) 8(1):e000696. 10.1136/jitc-2020-000696 32448804 PMC7252975

[18] Liang L Wen Y Hu R Wang L Xia Y Hu C. Safety and efficacy of PD-1 blockade-activated multiple antigen-specific cellular therapy alone or in combination with apatinib in patients with advanced solid tumors: a pooled analysis of two prospective trials. Cancer Immunol Immunother (2019) 68:1467–77. 10.1007/s00262-019-02375-z PMC11028216 31451841

[19] Xie L Xu J Sun X Guo W Gu J Liu K. Apatinib plus camrelizumab (anti-PD1 therapy, SHR-1210) for advanced osteosarcoma (APFAO) progressing after chemotherapy: a single-arm, open-label, phase 2 trial. J Immunother Cancer (2020) 8(1). 10.1136/jitc-2020-000798 PMC7223462 32376724

[20] Franses JW Hong TS Zhu AX. Nivolumab with gemcitabine plus cisplatin for biliary cancers: as easy as ABC? Lancet Gastroenterol Hepatol (2019) 4:575–7. 10.1016/S2468-1253(19)30148-7 31109809

[21] Ueno M Ikeda M Morizane C Kobayashi S Ohno I Kondo S. Nivolumab alone or in combination with cisplatin plus gemcitabine in Japanese patients with unresectable or recurrent biliary tract cancer: a non-randomised, multicentre, open-label, phase 1 study. Lancet Gastroenterol Hepatol (2019) 4:611–21. 10.1016/S2468-1253(19)30086-X 31109808

[22] Lin J Yang X Long J Zhao S Mao J Wang D. Pembrolizumab combined with lenvatinib as non-first-line therapy in patients with refractory biliary tract carcinoma. Hepatobiliary Surg Nutr (2020) 9(4):414–24. 10.21037/hbsn-20-338 PMC7423565 32832493

[23] Fontugne J Augustin J Pujals A Compagnon P Rousseau B Luciani A. PD-L1 expression in perihilar and intrahepatic cholangiocarcinoma. Oncotarget (2017) 8:24644–51. 10.18632/oncotarget.15602 PMC5421876 28445951

[24] Walter D Herrmann E Schnitzbauer AA Zeuzem S Hansmann ML Peveling-Oberhag J. PD-L1 expression in extrahepatic cholangiocarcinoma. Histopathology (2017) 71:383–92. 10.1111/his.13238 28419539

[25] Piha-Paul SA Oh DY Ueno M Malka D Chung HC Nagrial A. Efficacy and safety of pembrolizumab for the treatment of advanced biliary cancer: Results from the KEYNOTE-158 and KEYNOTE-028 studies. Int J Cancer (2020) 147:2190–8. 10.1002/ijc.33013 32359091

[26] Kim RD Chung V Alese OB El-Rayes BF Li D Al-Toubah TE. A Phase 2 Multi-institutional Study of Nivolumab for Patients With Advanced Refractory Biliary Tract Cancer. JAMA Oncol (2020) 6:1–8. 10.1001/jamaoncol.2020.0930 PMC7193528 32352498

[27] Yamashita S Passot G Aloia T Chun Y Javle M Lee J. Prognostic value of carbohydrate antigen 19-9 in patients undergoing resection of biliary tract cancer. J Br Surg (2017) 104:267–77. 10.1002/bjs.10415 28052308

[28] Nehls O Gregor M Klump B. Serum and bile markers for cholangiocarcinoma. Semin Liver Dis (2004) 24:139–54. 10.1055/s-2004-828891 15192787

[29] Shao F Zhang H Yang X Luo X Liu J. Adverse events and management of apatinib in patients with advanced or metastatic cancers: A review. Neoplasma (2020) 67:715–23. 10.4149/neo_2020_190801N701 32266817

[30] El-Khoueiry AB Sangro B Yau T Crocenzi TS Kudo M Hsu C. Nivolumab in patients with advanced hepatocellular carcinoma (CheckMate 040): an open-label, non-comparative, phase 1/2 dose escalation and expansion trial. Lancet (2017) 389:2492–502. 10.1016/S0140-6736(17)31046-2 PMC7539326 28434648

[31] Qin S Ren Z Meng Z Chen Z Chai X Xiong J. Camrelizumab in patients with previously treated advanced hepatocellular carcinoma: a multicentre, open-label, parallel-group, randomised, phase 2 trial. Lancet Oncol (2020) 21:571–80. 10.1016/S1470-2045(20)30011-5 32112738

