# Supplementary material for: The Efficacy and Safety of Apatinib Plus Camrelizumab in Patients With Previously Treated Advanced Biliary Tract Cancer: A Prospective Clinical Study
Source: Front Oncol. 2021 Apr 12;11:646979. doi: 10.3389/fonc.2021.646979 (PMC8071846; doi:10.3389/fonc.2021.646979)
Supplement: Supplementary file 1 [file Table_1.pdf]

Table 1. Comparison of efficacy and safety in different biliary tract cancer subtypes

Subgroup	ORR	PFS (months)	OS (months)	Grade 1-2 AEs	Grade 3-4 AES
All	19.0% (7-40)	4.4 (2.4-6.3)	13.1 (8.1-18.2)	100% (85.1-100)	63.6% (42.9-80.2)
ICC	20% (7-45.1)	4.4 (2.7-6.0)	11.9 (4.0-19.7)	100% (80.6-100)	68.7% (44.4-85.8)
ECC	0 (0-48.9)	3.2 (0-7.5)	15.6	100% (51.0-100)	50% (15-85)
GBC	50% (9.4-90.5)	2.7	2.8	100% (34.2-100)	50% (9.4-90.5)
$\chi 2$	2.666	-	-	-	0.649
p value*	0.264	0.958	0.725	-	0.723

ORR, objective response rate; PFS, progression-free survival; OS, overall survival; AEs, adverse events, ICC, intrahepatocellular carcinoma, ECC, extrahepatocellular carcinoma, GBC, gallbladder carcinoma

Table 2. Univariate and multivariate analysis of baseline characteristics for overall survival and progression-free survival

	Overall survival (OS)						Progression-free survival (PFS)					
	Univariate Cox analysis			Multivariate Cox analysis			Univariate Cox analysis			Multivariate Cox analysis		
**Variables**	**HR**	**95%CI**	***P***	**HR**	**95%CI**	***P***	**HR**	**95%CI**	***P***	**HR**	**95%CI**	***P***
Age (>60/<=60)	0.732	0.230-2.334	0.598				0.728	0.277-1.914	0.52			
Gender (female/male)	0.783	0.238-2.575	0.688				1.052	0.405-2.733	0.918			
HBV infection (yes/no)	1.498	0.463-4.843	0.5	1.121	0.255-4.933	0.88	1.186	0.435-3.235	0.739	0.921	0.290-2.927	0.889
ECOG PS (2/0-1)	2.12	0.453-9.932	0.34	5.091	0.735-35.271	0.099	0.724	0.165-3.178	0.669	0.944	0.199-4.477	0.942
Histopathologyical grade												
poorly differentiated/non-poorly	**2.79**	**0.824-9.444**	**0.099***	2.527	0.447-14.292	0.294	1.763	0.658-4.723	0.26	2.215	0.544-9.023	0.267
Recurrence after resection (yes/no)	1.516	0.448-5.123	0.503	1.857	0.385-8.954	0.441	1.265	0.479-3.341	0.635	1.789	0.536-5.968	0.344
Number of previous treatment												
after fist line/after second line	0.813	0.259-2.551	0.723	0.8	0.180-3.558	0.769	0.74	0.284-1.926	0.537	0.553	0.135-2.265	0.41
CA19-9 value (>=150)	0.416	0.117-1.480	0.176	0.53	0.083-3.374	0.501	0.616	0.236-1.608	0.323	1.378	0.296-6.425	0.683
Size of tumor (>=average value)	1.828	0.569-5.871	0.311	2.764	0.479-15.941	0.255	1.352	0.513-3.567	0.542	1.903	0.515-7.028	0.335
Site of metastases												
lung (yes/no)	**3.009**	**0.946-9.570**	**0.062***	2.48	0.493-12.483	0.271	1.357	0.510-3.611	0.541	1.308	0.383-4.468	0.669
intrahepatic (yes/no)	0.992	0.267-3.686	0.99				0.845	0.268-2.668	0.774			
lymph node (yes/no)	0.899	0.261-3.104	0.867				0.885	0.324-2.419	0.812			

HR, hazard rate; CI, confidence interval

Table 3. CA19-9 serum predicts the tumor size reduction

		CA19-9 decreased		
		+	-	sum
Tumor reduction	+	7	4	11
	-	3	6	9
Sensitivity	63.6%			
Specificity	66.7%			

Study Criteria

Inclusion Criteria:

1. Patients must have histologically or cytologically confirmed cholangiocarcinoma including intrahepatic cholangiocarcinoma, extrahepatic cholangiocarcinoma and gallbladder cancer. Patients with ampullary carcinoma are not eligible.

2. Patients must have failed or are intolerant to at least one line of systemic antitumor treatment including chemotherapy.

3. Patients who received adjuvant chemotherapy and had evidence of disease recurrence within 6 months of completion of the adjuvant treatment are also

eligible. If the patient received adjuvant treatment and had disease recurrence after 6 months, patients will only be eligible after failing or having intolerance to one line of systemic chemotherapy used to treat the disease recurrence.

4. Age ⩾ 18 years.

5. Eastern Cooperative Oncology Group (ECOG) Performance Status Assessment of 0-2.

6. Life expectancy of at least 12 weeks (3 months).

7. Subjects must be able to understand and be willing to sign the written informed consent form. A signed informed consent form must be appropriately obtained prior to the conduct of any trial-specific procedure. Subjects must be willing and able to comply with scheduled visits, treatment schedule, laboratory testing, and other study requirements.

8. All acute toxic effects of any prior treatment have resolved to NCI-CTCAE v4.0 Grade 1 or less at the time of signing the Informed Consent Form (ICF) except for alopecia.

9. For patients with advanced biliary tract cancer, liver function status Child-Pugh Class A or B (score<=7).

10. Adequate bone marrow, liver and liver function as assessed by the following laboratory requirements: Total bilirubin ⩽ 1.5 x the upper limits of normal (ULN), except for subjects with Gilbert Syndrome who can have bilirubin <3. Alanine aminotransferase (ALT) and aspartate amino-transferase (AST) ⩽ 2.5 x ULN (⩽ 5 x ULN for subjects with liver involvement of their cancer or stent placement).

Alkaline phosphastase limit ≤ 2.5 x ULN (≤ 5 x ULN for subjects with liver involvement of their cancer). Serum creatinine <2 x ULN. Hematologic parameters as follows: Platelet count ≥ 100,000 /mm3. Hemoglobin (Hb) ≥ 9 g/Dl. Absolute neutrophil count (ANC) ≥1000/mm. Blood transfusion to meet the inclusion criteria will be allowed.

11. Women of childbearing potential must have a negative serum or urine pregnancy test (minimum sensitivity of 25 IU/L or equivalent units of HCG) performed within24 hours prior to the start of treatment Post-menopausal women (defined as no menses for at least 1 year) and surgically sterilized women are not required to undergo a pregnancy test.

Subjects (men and women) of childbearing potential must agree to use adequate contraception beginning at the signing of the ICF until at least 3 months after the last dose of study drug. The definition of adequate contraception will be based on the judgment of the principal investigator or a designated associate.

12. Patients with history of hepatitis B and hepatitis C will be eligible but patients with hepatitis B must be started on antiviral therapy prior to beginning study therapy

13. Availability of archival tumor tissue for biomarkers analysis (FFPE block or cell block will be required). Specimen from primary site will be allowed. Patients must have at least 10 slides available. Repeat biopsy to obtain sufficient tissue for 10 slides is allowed.

Exclusion Criteria

1. Subjects with active CNS metastases are excluded. If CNS metastases are treated and subjects are at neurologic baseline for at least 2 weeks prior to enrollment, they will be eligible but will need a Brain MRI prior to enrollment. Subjects must be off corticosteroids or on a stable or decreasing dose of ≤ 10 mg daily prednisone (or equivalent).

2. Subjects with active, known or suspected autoimmune disease. Subjects with vitiligo, type I diabetes mellitus, residual hypothyroidism due to autoimmune thyroiditis only requiring hormone replacement, or conditions not expected to recur in the absence of an external trigger are permitted to enroll

3. Subjects with a condition requiring systemic treatment with either corticosteroids (>10 mg daily prednisone equivalent) or other immunosuppressive medications within 14 days of enrollment. Inhaled or topical steroids, and adrenal replacement steroid doses > 10 mg daily prednisone equivalent, are permitted in the absence of active autoimmune disease.

4. Previous or concurrent cancer within 3 years prior to treatment start EXCEPT for curatively treated cervical cancer in situ, non-melanoma skin cancer, superficial bladder tumors [Ta (non-invasive tumor), Tis (carcinoma in situ) and T1 (tumor invades lamina propria)].

5. Known history of human immunodeficiency virus (HIV) infection or acquired immunodeficiency syndrome (AIDS).

6. Child Pugh C disease

7. History of severe hypersensitivity reactions to other monoclonal antibodies

History of allergy or intolerance to study drug components or Polysorbate-80-containing infusions

8. Substance abuse, medical, psychological or social conditions that may interfere with the patient's participation in the study or evaluation of the study results.

History or concurrent condition of interstitial lung disease of any grade or severely impaired pulmonary function.

9. Unresolved toxicity higher than CTCAE grade 1 attributed to any prior therapy/procedure excluding alopecia.

10. Pregnant or breast-feeding patients. Women of childbearing potential must have a negative serum or urine pregnancy test (minimum sensitivity of 25 IU/L or equivalent units of HCG) performed within 24 hours prior to the start of treatment and a negative result must be documented before start of treatment.

11. Any illness or medical conditions that are unstable or could jeopardize the safety of the patient and his/her compliance in the study.

12. Anticancer chemotherapy during the study or within 4 weeks of study enrollment. Subjects must have recovered from the toxic effects of the previous anti-cancer chemotherapy (with the exception of alopecia). Anti-cancer therapy is defined as any agent or combination of agents with clinically proven anti-tumor activity administered by any route with the purpose of affecting the malignancy, either directly or indirectly, including palliative and therapeutic endpoints.

13. Hormonal therapy during the study or within 2 weeks of first study enrollment.

Investigational drug therapy outside of this trial during or within 4 weeks of first study treatment.

14. Notably, patients with severe esophageal varices or presented with positive fecal occult blood were also excluded.
